# Adipose tissue alleviates the stress response by releasing adiponectin during laparoscopic surgery in patients with colorectal cancer

**DOI:** 10.1186/s12944-021-01595-6

**Published:** 2021-11-20

**Authors:** Wenjiao Shi, Jian Lou, Xiaodan Zhang, Yun Ji, Xiaojian Weng, Jianer Du

**Affiliations:** 1 Department of Anesthesiology and SICU, Xinhua Hospital, School of Medicine, Shanghai Jiao Tong University, Kongjiang Road 1665, Shanghai, 200092 China; 2 Department of Intensive Care Unit, Shanghai General Hospital, School of Medicine, Shanghai Jiao Tong University, Shanghai, 200080 P. R. China

**Keywords:** Adiponectin, Adipose tissue, Laparoscopic surgery, Oxidative stress, Inflammatory response

## Abstract

**Background and objectives:**

Laparoscopic colorectal surgery causes a lower stress response than open surgery. Adiponectin is mainly derived from adipocytes and has antidiabetic, antioxidative, and anti-inflammatory capabilities. The objective of the present study was to investigate the protein expression of adiponectin in adipose tissue, and the serum levels of adiponectin, oxidative stress markers and proinflammatory factors during laparoscopic colorectal surgery and open surgery periods.

**Methods:**

Forty patients aged 60 to 80, with American Society of Anesthesiologists (ASA) I ~ II who underwent radical resection of colorectal cancer were recruited to the study. Laparoscopic group and open group included 20 patients each. Mesenteric adipose tissue and venous blood before (T1) and at the end (T2) of surgery were collected to examine adiponectin levels, and venous blood was collected to examine serum levels of oxidative stress related markers (superoxide dismutase (SOD), glutathione (GSH), lipid peroxide (LPO), malondialdehyde (MDA)), and inflammation-related factors (interleukin (IL)-1β, interleukin (IL)-6, tumor necrosis factor-α (TNF-α)).

**Results:**

Protein and serum levels of adiponectin were analyzed, and adiponectin levels were significantly increased at T2 than T1 in the laparoscopic surgery, while adiponectin levels were significantly higher in the laparoscopic surgery than in the open surgery at T2. In addition, the serum levels of SOD and GSH were significantly higher in the laparoscopic surgery than in open surgery at T2. However, the serum levels of LPO, TNF-α, IL-1β, and IL-6 were significantly lower in the laparoscopic group than in open group at T2.

**Conclusion:**

Laparoscopic surgery induced higher levels of adiponectin in both adipose tissue and the bloodstream. Oxidative stress and the inflammatory response were lower during laparoscopic colorectal surgery than during conventional open surgery. These data suggest that adipose tissue may alleviate the stress response during laparoscopic surgery by releasing adiponectin in patients with colorectal cancer.

## Introduction

Colorectal cancer has attracted in recent years increasing attention because of its high morbidity and mortality, and radical resection of colorectal cancer remains the most effective treatment [1]. Although various surgical methods have already been proposed, laparoscopic surgery is becoming increasingly popular among surgeons and patients because compared to open surgery, it is less invasive, recovery is quick, and blood loss and operative injury are reduced [2]. However, it has also been reported that laparoscopic surgery causes a systemic stress response [3]. Surgical operations are known to induce sophisticated physiological stress responses that involves the activation of metabolic, endocrine, inflammatory and immunological mediators [4]. However, the degree to which this response is harmful remains unclear.

Surgical trauma causes stress responses such as inflammatory response and oxidative stress [5]. Oxidative stress occurs when the generation of reactive oxygen species (ROS) and antioxidants in the body is out of balance [6]. Excessive production of ROS is detrimental to cellular components including nucleic acids, proteins, and lipids. Abdominal surgery always causes oxidative stress. Pappas-Gogos et al. reported that laparoscopic colorectal surgery was associated with lower oxidative stress than open surgery by examining oxidative stress markers including 8-hydroxyguanosine, 8-isoprostanes, protein carbonyl, and 3-nitrotyrosine [7]. Surgery can produce proinflammatory cytokines and severe immune system changes, leading to an increased incidence of postoperative sepsis and inflammatory complications [5]. A previous study indicated that rectal surgery caused the release of pro- and anti-inflammatory cytokines, and there was a lower inflammatory response during the laparoscopic surgery than open surgery [8]. However, no studies have shown the possible regulatory factors associated with the reductions in the inflammatory response and oxidative stress during laparoscopic surgery.

Adiponectin is an important adipokine that is secreted mainly by adipocytes and has anti-inflammatory, antioxidative, antidiabetic and antiatherosclerotic properties [6]. As endocrine cells, adipocytes are capable of releasing a large number of biologically active molecules into the circulation, thus participate in the communication network between adipose tissue and other organs [9]. Recently, clinical studies have reported that the increased malignancy in colon cancer is related to lower levels of adiponectin in serum [10]. In addition, in colorectal cancer surgery, low preoperative adiponectin levels are associated with an increased incidence of postoperative inflammatory damage and complications such as sepsis [11]. Thus, it has been hypothesized that high preoperative adiponectin levels in the circulation may protect surgical patients from inflammation-related postoperative complications [12]. However, the changes in serum adiponectin levels during laparoscopic surgery and open surgery, and whether serum adiponectin levels are derived from adipose tissue remain unclear. Several studies have shown that adiponectin has a protective effect on different animal models by decreasing oxidative stress and inflammation levels [6, 13]. Thus, we hypothesized that lower levels of oxidative stress and inflammatory factors after laparoscopic surgery compared with open surgery may be related to increased circulating levels of adiponectin released from adipose tissue.

The objective of the present study was to compare adiponectin levels in both the bloodstream and adipose tissue between laparoscopic and open groups in colorectal cancer patients. In addition, we explored whether laparoscopic colorectal surgery caused a less pronounced release of oxidative stress markers and proinflammatory cytokines than open surgery.

## Materials and methods

### Patients

This study was performed at Shanghai Jiao Tong University School of Medicine Affiliated Xinhua Hospital. Forty colorectal cancer patients aged 60 to 80 with American Society of Anesthesiologists (ASA) classification I ~ II who were selected to undergo radical resection of colorectal cancer were enrolled in this study (registration number: ChiCTR2000036910). Among these patients, twenty patients enrolled in laparoscopic surgery group, and twenty patients enrolled in open surgery group. Approval of the Ethics Committee of Shanghai Jiao Tong University School of Medicine Affiliated Xinhua Hospital (approval number: XHEC-SHHDC-2020-049) and individual informed consent were obtained. The exclusion criteria were as follows: age < 60 years or ≥ 80; body mass index (BMI) ≥28; severe cardiopulmonary insufficiency; fever; infection; and known autoimmune disease, diabetes, uncontrolled hypertension, alcohol or drug abuse. The study was conducted from January 2021 to June 2021. All surgeries were performed by the same surgical team.

### Anesthesia

None of the patients received special preoperative medication. Patients fasted overnight before arriving in the operating room. Endotracheal intubation was performed under general anesthesia. Before the induction of anesthesia, standard monitors were applied to record arterial blood pressure and electrocardiogram. A bispectral index system (BIS) was used to monitor the depth of anesthesia. General anesthesia was induced with propofol (2 mg/kg), remifentanil (1 μg/kg), and cisatracurium besilate (0.2 mg/kg). Ventilation was controlled with a tidal volume of 10 ml/kg, and the respiratory rate was adjusted to maintain a carbon dioxide end-tidal concentration of 35–40 mmHg. Sevoflurane, remifentanil and propofol were used to maintain anesthesia with a BIS score of 45–55. In addition, rocuronium was used to maintain muscular blockade. Pneumoperitoneum of 12–15 mmHg was maintained in the laparoscopic surgery group throughout the operation.

### Sample collection

Blood samples were collected from peripheral veins. White adipose tissue was collected from mesenteric adipose tissue. Adipose tissues and blood samples were collected at two different times. The first samples were collected at the time at which the abdominal cavity was entered (T1). The second samples were obtained 5 min after pneumoperitoneum deflation (laparoscopic surgery) or 5 min after the distal division of the colorectal tumor (open surgery) (T2). The laparoscopic group maintained a pneumoperitoneum pressure of 12–15 mmHg throughout the operation. Six milliliters of venous blood were collected from each patient in a coagulation tube for serum separation. The bloods samples were allowed to naturally coagulate for 10 to 20 min at room temperature. Then, centrifuged for 10 min at 10,000×g and 4 °C. The upper serum layer was carefully collected and stored in an ultralow-temperature freezer at − 80 °C for use. The adipose tissues were immediately placed into liquid nitrogen after they were removed from the body and then stored in a − 80 °C freezer for later use.

### Measurement of adiponectin

Western blotting analysis was performed to examine the protein expression of adiponectin in adipose tissue. Frozen adipose tissue (100 mg) was homogenized in precooled lysis buffer (Beyotime Biotechnology Co., Ltd., Shanghai, China). Then, the supernatant was collected after the homogenate was centrifuged at 14,000×g for 10 min at 4 °C. A BCA protein assay kit was used to measure the protein concentration (Beyotime Biotechnology Co., Ltd., Shanghai, China). After being boiled for 10 min at 100 °C, the samples were loaded for 10% polyacrylamide gel electrophoresis and then transferred to polyvinylidene difluoride membranes. The membranes were blocked in 5% nonfat milk for 1 h at room temperature and then incubated with primary antibodies against adiponectin (1:2000, Abcam, Cambridge, MA) overnight at 4 °C. The membranes were washed three times in TBST and then incubated with secondary horseradish peroxidase-conjugated anti-rabbit IgG (Beyotime Biotechnology) for 1 h at room temperature. Proteins were visualized by ECL reagent (Beyotime Biotechnology) and quantified using Image Lab 5.0 software (Bio-Rad Laboratories, USA). All of the membranes were reblotted with an antibody against β-actin (1:2000, BOSTER) after being stripped to verify the uniformity of protein loading and the transfer efficiency across the test samples.

The serum level of adiponectin was measured with an enzyme-linked immunosorbent assay (ELISA) kit (CUSABIO, Wuhan Huamei Biotech Co., Ltd. Wuhan, China). Samples and controls were incubated for 2 h at 37 °C on the prepared plates. Then, the liquid was removed, and 100 μl of biotin-antibody was added to each well and incubated for 1 h at 37 °C. Each well was washed three times. Then, HRP-avidin was added to each well and incubated for 1 h at 37 °C. The plates were again washed five times before TMB substrate was added and incubated for 15–30 min at 37 °C in the dark. After the stop solution was added, the microplates were analyzed on a microplate reader within 5 min and the wavelength was set at 450 nm. Values were estimated by the ELISA data analysis software Curve Expert (version: 1.4), which is capable of generating a four-parameter logistic (4-PL) curve-fit.

### Detection of oxidative stress related markers (GSH, SOD, LPO, MDA)

Oxidative stress status was assessed by estimating serum levels of glutathione (GSH), superoxide dismutase (SOD), lipid peroxide (LPO), and malondialdehyde (MDA). GSH levels were determined by a GSH assay kit (microplate method). Serum SOD levels were determined by a SOD assay kit (WST-1 method) and LPO levels were estimated by a lipid peroxidation assay kit. Serum MDA levels were estimated by an MDA assay kit (thibabituric acid method). All of the assay kits were purchased from Nanjing Jiancheng (Nanjing Built Technology Co. Ltd., Jiangsu, China) and performed follow the manufacturer’s instructions.

### Detection of proinflammatory factors (TNF-α, IL-1β, IL-6)

Serum levels of IL-1β, IL-6 and TNF-α were measured with ELISA kits according to the manufacturer’s instructions (CUSABIO, Wuhan Huamei Biotech Co., Ltd. Wuhan, China). The detailed procedures were the same as those described above.

### Statistical analysis

Adiponectin serum levels were used to evaluate the sample size. The sample size was calculated to detect a 25% relative difference in adiponectin between groups on the basis of the postoperative response of circulating adiponectin according to a previous study by Chelazzi and colleagues [14]. Our hypothesis was that there would be a 25% (SD 10%) relative difference in adiponectin between the laparoscopic and open groups, which meant that a sample size of 20 patients in each group was needed to obtain a power of 80% for a significance level of 5% with a two-tailed test.

Continuous variables with a Gaussian distribution are presented as the mean ± standard deviation (SD) as specified. Continuous variables with no Gaussian distribution are presented as the median (IQR). The Komolgorov-Smirnov test was used to check the assumption of normality. For multiple comparisons of normally distributed variables between more than two groups, one-way analysis of variance (ANOVA) was used with post- hoc t tests and Bonferroni corrections. The differences in patient characteristics between the two groups were assessed using χ2 (sex), Student’s t test (age and BMI), and the Mann–Whitney U-test (duration of surgery, blood loss, and hospital stay after the surgery). All tests were two-tailed, and *P* < 0.05 was considered statistically significant. The SPSS version 20.0 (SPSS Inc., Chicago, IL) was used throughout.

## Results

The patient characteristics of each group are shown in Table 1. There was no significant difference between groups concerning age, sex, BMI, or hospital stay after the surgery. There was a significant difference concerning blood loss and duration of surgery. The median bleeding volume was 30 ml in the laparoscopic group and 90 ml in the open group. The median duration of surgery was 352 min in the laparoscopic group and 253 min in the open group.

**Table 1:** Patient characteristics in the laparoscopic and open groups

	Laparoscopic surgery	Open surgery	*P* value
N	20	20	
Gender (F/M)	9/11	11/9	0.752
Age (mean ± SD) (years)	65.95 ± 5.95	68.95 ± 7.99	0.186
BMI (mean ± SD) (kg/m^2^)	22.64 ± 3.12	22.71 ± 2.25	0.939
Duration of surgery (median [IQR]) (min)	352 [320–393]	253 [232–283]	< 0.005
Blood loss (median [IQR]) (ml)	30 [30–50]	90 [52.5–100]	0.003
Hospital stay after the surgery (median [IQR]) (days)	16 [15–17]	16 [14.25–18]	0.556

*SD* standard deviation, *IQR* interquartile range

### Adiponectin levels

**Fig. 1:**
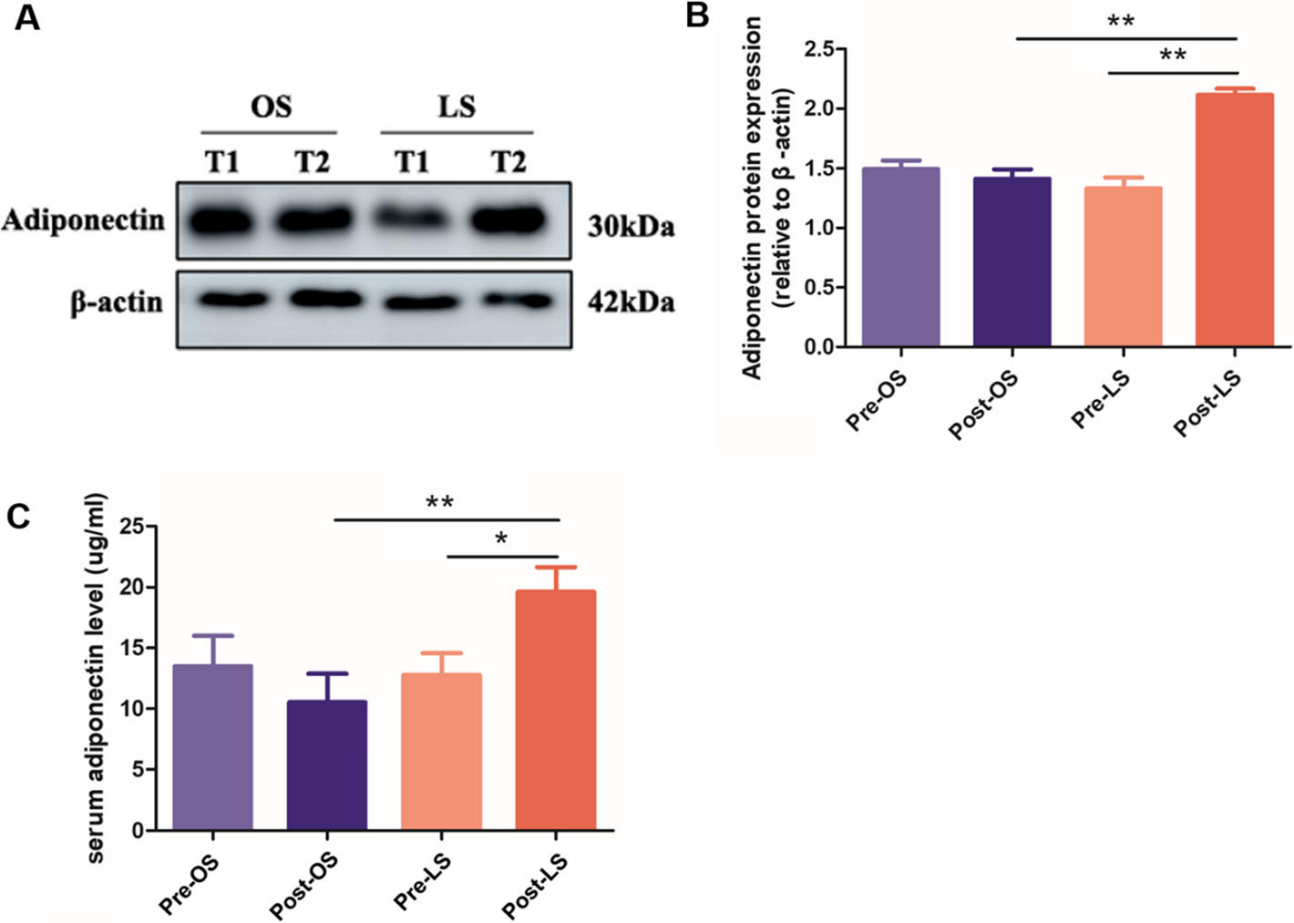
Adiponectin levels in the laparoscopic and open surgery groups. **A**: Representative blot. Equal protein loading was confirmed with β-actin. **B**: Densitometric analysis of adiponectin protein expression. **C**: Serum level of adiponectin. Pre-LS: prelaparoscopic surgery; Post-LS: postlaparoscopic surgery; Pre-OS: preopen surgery; Post-OS: postopen surgery. The data are expressed as the mean ± SD (*n* = 20). *P < 0.05 and **P < 0.005

At T1 (the time at which the abdominal cavity was entered), there were no notable differences between the laparoscopic and open groups regarding adiponectin levels in either serum or adipose tissue. ELISA and western blotting showed that the concentrations of serum adiponectin were significantly higher in the laparoscopic group than the open group at T2. In addition, the adiponectin levels were notably increased in the laparoscopic surgery at T2 than T1. There was no significant difference between T1 and T2 in the open group (Fig. 1).

### Serum levels of GSH, SOD, LPO, and MDA

**Fig. 2:**
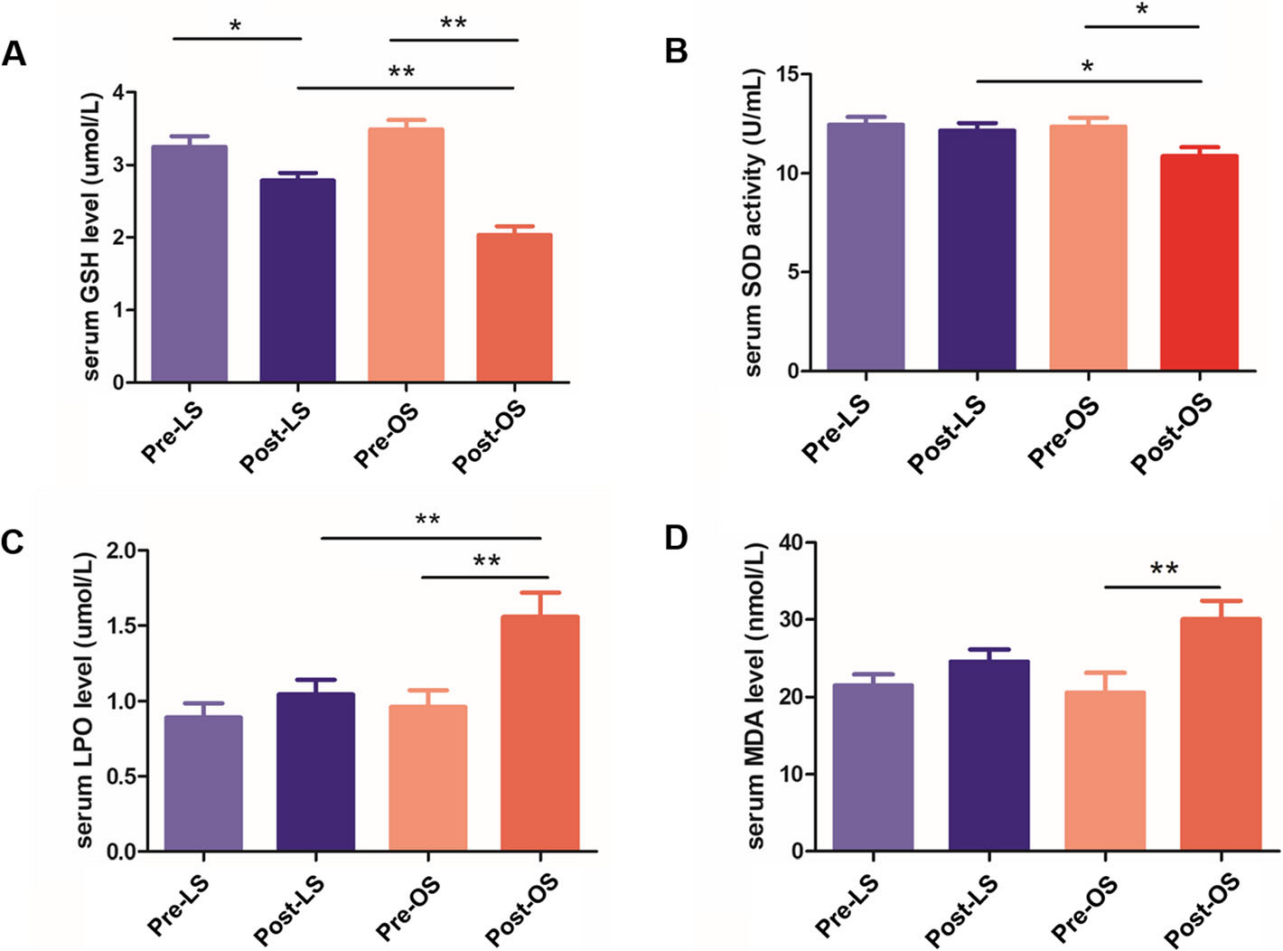
Serum levels of glutathione (GSH), superoxide dismutase (SOD), lipid peroxide (LPO), and malondialdehyde (MDA) in the laparoscopic and open surgery groups. **A**: Comparison of serum levels of GSH. **B**: Comparison of serum levels of SOD. **C**: Comparison of serum levels of LPO. **D**: Comparison of serum levels of MDA. Pre-LS: prelaparoscopic surgery; Post-LS: postlaparoscopic surgery; Pre-OS: preopen surgery; Post-OS: postopen surgery. The data are expressed as the mean ± SD (*n* = 20). *P < 0.05 and **P < 0.005

There was no significant difference between the laparoscopic and open groups regarding serum levels of GSH, SOD, LPO, or MDA at T1. At T2, serum GSH levels were significantly decreased than those at T1 in both the laparoscopic and open groups, serum SOD levels were significantly decreased in the open group compared to those at T1, and serum LPO and MDA levels were significantly increased in the open group compared to those at T1. In addition, there were significantly higher GSH and SOD levels but lower LPO levels in the laparoscopic group than in the open group at T2. These data suggested that oxidative stress was lower during laparoscopic surgery than during conventional open surgery (Fig. 2).

### Serum levels of IL-1β, IL-6, and TNF-α

**Fig. 3:**
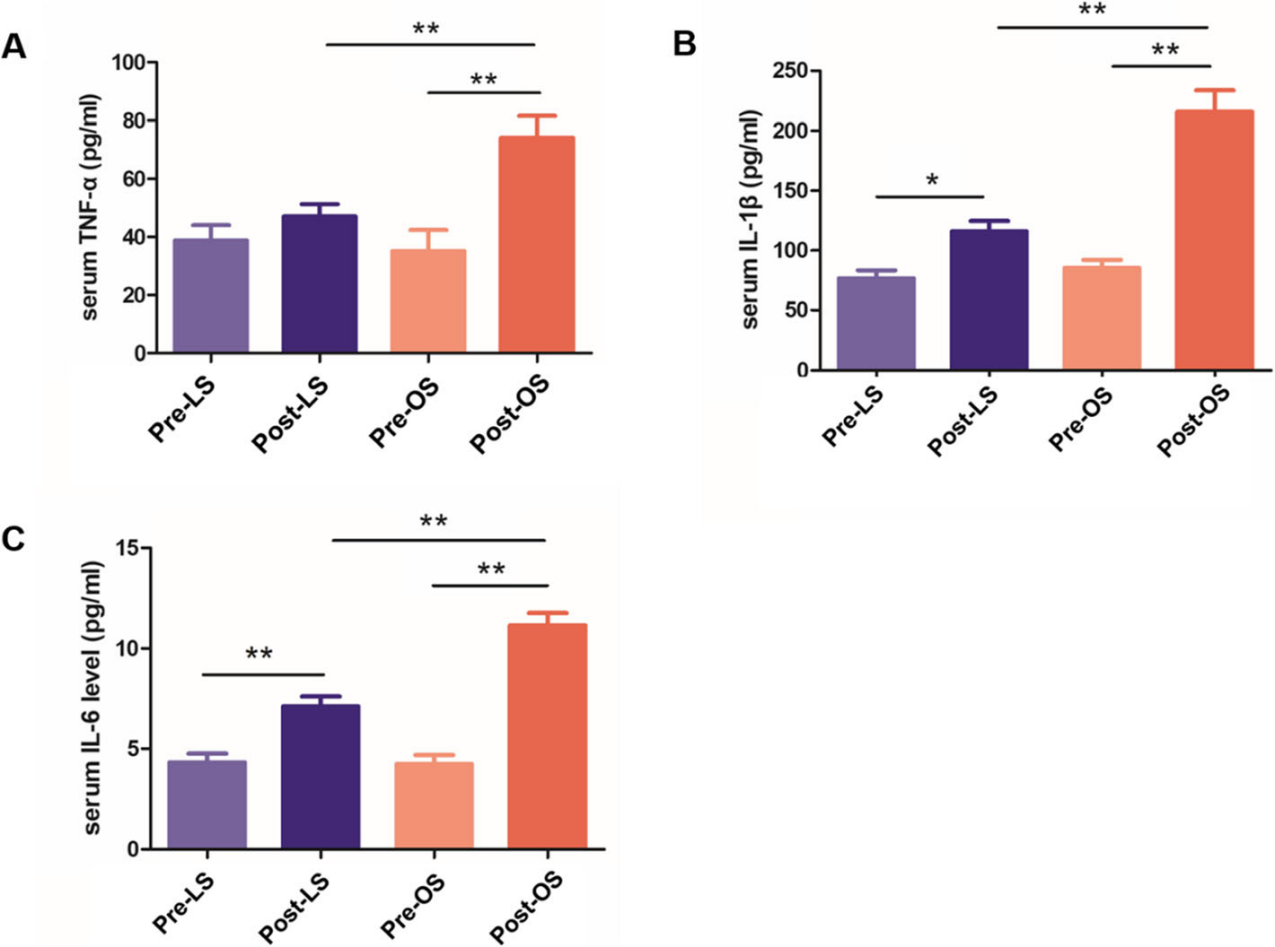
Serum levels of tumor necrosis factor-α (TNF-α), interleukin (IL)-1β, and interleukin (IL)-6 in the laparoscopic and open surgery groups. **A**: Comparison of serum levels of TNF-α. **B**: Comparison of serum levels of IL-1β. **C**: Comparison of serum levels of IL-6. Pre-LS: prelaparoscopic surgery; Post-LS: postlaparoscopic surgery; Pre-OS: preopen surgery; Post-OS: postopen surgery. The data are expressed as the mean ± SD (*n* = 20). *P < 0.05 and **P < 0.005

There was no significant difference between the laparoscopic and open groups regarding serum levels of TNF-α, IL-1β or IL-6 at T1. At T2, serum IL-1β and IL-6 levels were significantly increased compared to those at T1 in both the laparoscopic and open groups, and serum TNF-α levels were significantly increased in the open group than those at T1. In addition, there were significantly lower IL-1β, IL-6 and TNF-α levels in the laparoscopic group than in the open group at T2. These results suggested that the inflammatory response was lower during laparoscopic surgery than during conventional open surgery (Fig. 3).

## Discussion

The present study shows that the protein expression levels of adiponectin in mesenteric adipose tissue are significantly upregulated accompanied by an increase in serum adiponectin levels during laparoscopic colorectal surgery than conventional open surgery. In addition, this study also shows that there are significant reductions in oxidative stress (as indicated by lower levels of LPO and MDA and higher levels of GSH and SOD) and the inflammatory response (as indicated by lower levels of IL-6, IL-1β, and TNF-α) during laparoscopic surgery compared to open surgery in colorectal cancer patients. Taken together, these results suggest that the reductions in oxidative stress and inflammatory response in laparoscopic colorectal surgery may be due to the increases in circulating adiponectin levels produced by white adipose tissue. Adipose tissue plays an important role during the laparoscopic surgery period.

Adipose tissue is a generally acknowledged endocrine organ and has the ability to secrete a large number of endocrine factors that manage various physiological functions [9]. Adipose tissues is divided into white adipose tissue (WAT), which is the site of energy storage, and brown adipose tissue, which functions nonshivering thermogenesis particularly in small mammals and human neonates [15]. The WAT obtained in this study was mesenteric adipose tissue in the distal mesentery site far from the tumor. WAT contains a variety of cell types, predominantly adipocytes [16]. As endocrine cells, adipocytes establishes a communication network between WAT and other organs via secreting adipokines into circulation [9]. Adiponectin is an important adipokine that is produced mostly by adipocytes. This study showed that the protein expression of adiponectin in WAT was significantly increased in the laparoscopic group than the open group after surgery, accompanied by significantly increased serum adiponectin levels. The results indicated that pneumoperitoneum may cause metabolic changes in WAT. The most plausible explanation is that increased intra-abdominal pressure and inflation-deflation may induce adipose tissue to produce adiponectin and release it into circulation. To our knowledge, this is the first study to find that long-term pneumoperitoneum during surgery increased the protein expression of adiponectin in adipose tissue.

Many studies have assessed the association of the risk of colorectal cancer with circulating adiponectin levels [17–19]. However, the conclusions of these epidemiological researches have been discrepant. Nevertheless, most researches suggest that adiponectin levels in circulation is inversely associated with the risk of colorectal cancer. A prospective study suggested that in colorectal cancer patients, prediagnostic plasma adiponectin was associated with an increased risk of colorectal cancer-specific and overall mortality, and was more obvious in those with metastatic disease [20]. However, changes in serum adiponectin levels among colorectal cancer patients who undergo laparoscopic or open surgery remain unknown. The data in this study showed that adiponectin levels increased prominently after laparoscopic surgery compared to open surgery, indicating that laparoscopic surgery exerts a protective effect on the body, and the increased adiponectin levels were likely due to pneumoperitoneum increasing abdominal pressure and activating adipose tissue to release adiponectin.

Colorectal operation causes the secretion of both pro- and anti-inflammatory factors. Recent studies have shown that the laparoscopic rectal surgery induces lower inflammatory response than conventional open surgery [8, 21]. Our findings of a reduction in the postoperative release of TNF-1α, IL-1β and IL-6 during laparoscopic surgery compared to open confirmed the conclusions of these studies. IL-6 acts as a major cytokine has both pro-inflammatory and anti-inflammatory effects and is involved in the stress response to surgical trauma, sepsis, and burns [22]. Surgical trauma can induce increased IL-6 levels in plasma, and elevated IL-6 levels may predict surgical complications that result in peritonitis [23]. TNF-α and IL-1β are both proinflammatory cytokines and can induce an acute phase reaction with local and systemic inflammation [24]. Thus, lower levels of TNF-1α, IL-1β and IL-6 after laparoscopic surgery may predict better prognosis and fewer surgical complications. However, no studies have shown how TNF-1α, IL-1β and IL-6 levels decrease during laparoscopic surgery. A previous study reported that adiponectin could reduce inflammation by suppressing macrophage differentiation, changing the macrophage phenotype to an anti-inflammatory state, and reducing the expression of Toll-like receptor 4 [25]. Adiponectin has a protective effect on the vasculature and colon by suppressing inflammation [26]. These data indicated lower TNF-1α, IL-1β and IL-6 levels in laparoscopic surgery, which were probably due to the anti-inflammatory effects of adiponectin.

Surgical trauma causes increased oxidative stress response [27]. However, inconsistent results were revealed in a new systematic review of studies comparing oxidative stress in laparoscopic and open surgery [28]. Inconsistency in results may be due to the heterogeneity in study design, inclusion criteria, differences in biomarkers selection and the time point. The majority of studies report that oxidative stress is increased in the open surgery [29, 30]. Consistent with previous studies, we also confirmed that oxidative stress was lower during laparoscopic surgery than during open surgery, which as by increased GSH and SOD levels and decreased LPO and MDA levels. GSH and SOD are important antioxidants in vivo that can participate in multifaceted physiological activities [31]. LPO and MDA are end products of enzymatic and nonenzymatic lipid peroxidation. MDA, which is the most important product of cellular lipid oxidation, can exacerbate damage to the cell membrane and reflect the extent of damage. The reaction of saturated fatty acids with oxygen free radicals generates LPO. LPO acts as a cytotoxic peroxide can damage biofilms and cellular functions [32]. Based on this research and those of previous studies, the hypothesis that the reduction in oxidative stress during laparoscopic surgery may be due to the antioxidative effect of adiponectin is reasonable.

### Strengths and limitations

The present study has several strengths. First, to our knowledge, this is the first study to examine the protein expression of adiponectin in mesenteric adipose tissue during laparoscopic and open surgery. The results revealed that the protein expression and serum concentration of adiponectin were notably increased during laparoscopic surgery than open surgery. Furthermore, the results showed that the laparoscopic surgery significantly alleviated oxidative stress and the inflammatory response compared to open surgery. These data strengthen the hypothesis that upregulated adiponectin expression in adipose tissue exerts a protective effect on patients undergoing laparoscopic surgery by alleviating oxidative stress and the inflammatory response.

There are certain limitations that should be noted. This study just examined changes in adiponectin, oxidative stress, and inflammatory factors during the pre- and postoperative periods. However, other times should be selected to observe the changes in these biomarkers. Longterm follow-up is needed to analyze whether elevated adiponectin levels during laparoscopic surgery improve patient outcomes. This limitation was mainly due to the difficulty of obtaining mesenteric adipose tissue after surgery. The sample size was not sufficient to perform the regression analysis; thus, it is needed to expand the sample size in a subsequent study and preform logistic regression to evaluate if laparoscopic colorectal surgery really alleviates stress response and perform KM and COX regression to evaluate the difference between laparoscopic colorectal surgery and open surgery. In addition, this study did not deeply investigate the mechanism by which adiponectin downregulated oxidative stress and the inflammatory response to alleviate surgical damage. This topic will be further studied in clinical or animal studies.

## Conclusion

In conclusion, this study revealed that laparoscopic surgery upregulated the protein expression of adiponectin in mesenteric adipose tissue and significantly increased serum adiponectin levels while significantly decreased oxidative stress and the inflammatory response compared to open surgery. In clinical practice, adiponectin may be used to predict and prevent oxidative stress and inflammation. These results may provide a reference for surgeons to customize surgical procedures for patients with colorectal cancer. To reduce the complications of open surgery, pursuing a higher adiponectin level may be appropriate. In the future, prospective studies with more comprehensive data and larger sample sizes are needed to confirm the role of adiponectin during laparoscopic surgery.

## Data Availability

The data that support the findings of this study are available from the corresponding author upon reasonable request.

## Abbreviations

ASA: American Society of Anesthesiologists
SOD: Superoxide dismutase
GSH: Glutathione
LPO: Lipid peroxide
TNF-α: Tumor necrosis factor-α
MDA: Malondialdehyde
IL-6: Interleukin − 6
ROS: Reactive oxygen species
IL-1β: Interleukin -1β
BIS: Bispectral index system
WAT: White adipose tissue
BMI: Body mass index
